# Cembranoids from the Dongsha Atoll Soft Coral *Lobophytum crassum*

**DOI:** 10.3390/md9122705

**Published:** 2011-12-15

**Authors:** Shih-Tseng Lin, Shang-Kwei Wang, Chang-Yih Duh

**Affiliations:** 1 Department of Marine Biotechnology and Resources, National Sun Yat-sen University, Kaohsiung 804, Taiwan; Email: m975020026@student.nsysu.edu.tw; 2 Department of Microbiology, Kaohsiung Medical University, Kaohsiung 807, Taiwan; 3 Asia-Pacific Ocean Research Center, National Sun Yat-sen University, Kaohsiung 804, Taiwan

**Keywords:** soft coral, *Lobophytum crassum*, cytotoxicity

## Abstract

Chemical investigation of the Dongsha Atoll soft coral *Lobophytum crassum* has afforded four new cembranoids, crassumols A–C (**1**–**3**) and 13-acetoxysarcophytoxide (**4**). The structures of these isolated compounds were elucidated by extensive NMR and HRESIMS experiments. The cytotoxicity and anti-HCMV (Human cytomegalovirus) activities of **1**–**4** were evaluated *in vitro*. Compound **4** exhibited cytotoxicity against A-549 (human lung carcinoma) cell line with an ED_50_ of 3.6 μg/mL.

## 1. Introduction

**Figure 1:**
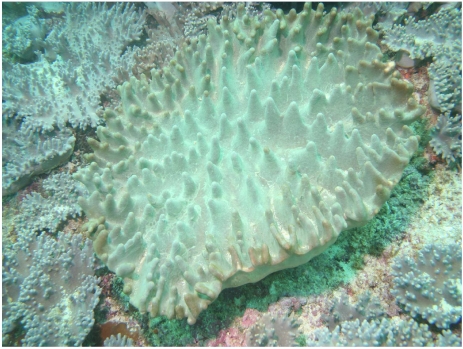
Soft coral *Lobophytum crassum*.

Soft corals belonging to the genus *Lobophytum* (Alcyoniidae) have been well recognized as a rich source of secondary metabolites endowed with a range of structural diversity and various biological activities [1,2,3,4,5,6,7,8,9,10,11,12,13,14,15,16,17,18,19,20,21]. Previous bioassay results of some cembraniods and their analogues have demonstrated remarkable pharmacological potential such as *in vitro* cytotoxicity against various cancer cell lines [2,3,4,5,6,7,8,9], anti-inflammatory properties [10,11,12], antimicrobial activities [10], and HIV-inhibitory activity [13]. In view of their scientific significance and potential usage, we have investigated the Dongsha Atoll soft coral *Lobophytum crassum* (Von Marenzeller, 1886) (Figure 1) for the bioactive substances. Four new cembranoids, crassumols A–C (**1**–**3**) and 13-acetoxysarcophytoxide (**4**) were isolated from the acetone-soluble of Dongsha Atoll collection of *L. crassum*.

## 2. Results and Discussion

**Figure 2:**
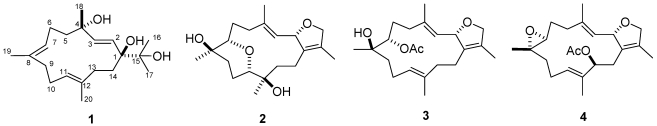
The structures of compounds **1**–**4**.

Specimens of *L. crassum* were frozen immediately after collection. Conventional extraction procedures were used, and the acetone extract was exhaustively partitioned between EtOAc and H_2_O to afford the EtOAc-soluble fraction, which was evaporated under vacuum to yield a dark brown gum (20 g). The resultant concentrated residue was subsequently subjected to fractionation with column chromatography and high-performance liquid chromatography, leading to the isolation of four new cembranoids, crassumols A–C (**1**–**3**) and 13-acetoxysarcophytoxide (**4**) (Figure 2). 

**Figure 3:**
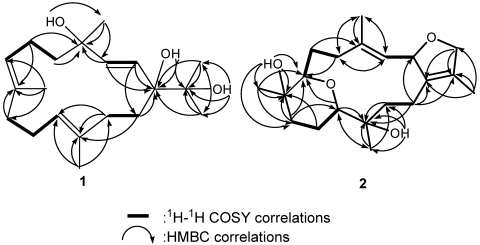
Selected ^1^H–^1^H COSY (▬) and HMBC (→) correlations of **1** and **2**.

**Figure 4:**
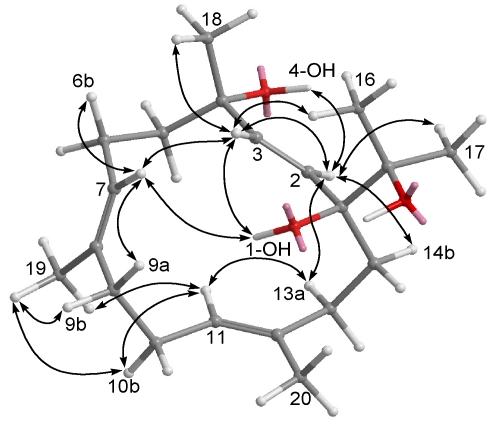
Selected NOESY correlations of **1**.

Crassumol A (**1**) appeared as colorless oil. Its HRESIMS (*m/z* 345.2408, [M + Na]^+^) and NMR spectroscopic data (Table 1 and Table 2) established the molecular formula C_20_H_34_O_3_, implying the existence of four degrees of unsaturation. Three tertiary hydroxyls were recognized as being present in **1** from its ^13^C NMR signals at *δ*_C_ 81.5 (qC, C-1), 73.7 (qC, C-4), and 75.1 (qC, C-15) and ^1^H NMR signals at *δ*_H_ 2.52 (1H, s, OH-1), 3.58 (1H, s, OH-4), and 3.03 (1H, s, OH-15) as well as from a broad IR absorption at 3421 cm^−1^. Moreover, the ^13^C NMR signals at *δ*_C_ 128.3 (CH, C-2), 139.1 (CH, C-3), 129.0 (CH, C-7), 133.2 (qC, C-8), 126.3 (CH, C-11), and 137.0 (qC, C-12), assigned one disubstituted double bond and two trisubstituted double bonds in **1**. The above functionalities also account for three of the four degrees of unsaturation, suggesting a monocyclic structure in **1**. By interpretation of ^1^H–^1^H COSY correlations, it was possible to establish four partial structures from H-2 to H-3, from H_2_-5 to H-7, from H-9 to H-11, and from H-13 to H-14 (Figure 3). Moreover, the connectivities of these partial structures through the quaternary carbons were further established by the HMBC correlations (Figure 3). Thus, the gross structure of crassumol A was assigned as **1**. The relative configurations of **1** were assigned by NOESY correlations (Figure 4) between H-3/Me-18, H-3/OH-1, H-3/H-7, H-2/OH-4, H-2/H-13a, H-11/H-13a, and Me-19/H-11. On the basis of the aforementioned observations, the structure of crassumol A (**1**) was unambiguously established as (1S*,4S*,2*E*,7*E*,11*E*)-1,4,15-trihydroxycembra-2,7,11-trien.

**Table 1:** ^1^H NMR data *^a^* for compounds **1**–**4**.

	1	2	3	4
2	5.79 d (15.6) *^b^*	5.51 d (9.6)	5.53 d (10.4)	5.60 d (11.8)
3	5.92 d (15.6)	5.59 d (9.6)	5.03 d (10.4)	5.23 d (11.8)
5a	1.94 ddd (13.2, 8.0, 2.0)	2.48 m	2.15 m	2.34 m
5b	1.67 m	2.30 m	1.93 m	2.36 m
6a	1.67 m	2.42 m	1.92 m	2.96 m
6b	2.27 m	1.84 m	1.82 m	1.63 m
7	5.32 t (8.0)	3.53 dd (10.8, 2.8)	5.04 d (10.0)	2.59 t (4.0)
9a	2.13 m	2.16 m	1.87 m	2.15 ddd (13.2, 4.8, 2.8)
9b	1.97 m	2.00 m	1.60 m	0.92 td (13.2, 3.2)
10a	2.06 m	2.20 m	2.23 m	2.27 m
10b	2.08 m	1.78 m	2.06 m	1.92 m
11	5.22 dd (9.4, 4.8)	3.74 d (12.0)	4.89 br d (8.0)	5.41 d (10.8, 5.2)
13a	2.22 m	2.22 m	1.97 m	5.16 d (9.2)
13b	2.07 m	1.78 m	1.87 m	
14a	2.12 m	2.64 dt (13.2, 9.8)	2.57 ddd (14.0, 11.6, 8.0)	2.75 dd (14.8, 10.4)
14b	1.71 m	2.07 m	2.14 m	1.87 m
16	1.07 s	4.57 dq (15.2, 4.8)	4.48 m	4.49 br s
17	1.12 s	1.59 s	1.65 s	1.66 s
18	1.25 s	1.82 s	1.87 s	1.91 s
19	1.59 s	1.45 s	1.09 s	1.28 s
20	1.64 s	1.48 s	1.65 s	1.61 s
OH-1	2.52 s			
OH-4	3.58 s			
OH-15	3.03 s			
OAc			2.11 s	2.00 s

*^a^* Spectra were measured in CDCl_3_ (400 MHz); *^b^* *J* values (in Hz) in parentheses.

**Table 2:** ^13^C NMR data *^a^* of compounds **1**–**4**.

	1	2	3	4
1	81.5 (qC) *^b^*	126.8 (qC)	134.1 (qC)	130.8 (qC)
2	128.3 (CH)	85.1 (CH)	83.8 (CH)	83.3 (CH)
3	139.1 (CH)	126.7 (CH)	127.6 (CH)	126.1 (CH)
4	73.7 (qC)	137.6 (qC)	137.9 (qC)	140.2 (qC)
5	45.3 (CH_2_)	35.8 (CH_2_)	35.6 (CH_2_)	37.7 (CH_2_)
6	24.1 (CH_2_)	24.2 (CH_2_)	24.8 (CH_2_)	25.1 (CH_2_)
7	129.0 (CH)	83.7 (CH)	77.3 (CH)	61.7 (CH)
8	133.2 (qC)	68.3 (qC)	75.3 (qC)	59.5 (qC)
9	39.9 (CH_2_)	40.9 (CH_2_)	37.4 (CH_2_)	39.7 (CH_2_)
10	24.4 (CH_2_)	23.2 (CH_2_)	23.7 (CH_2_)	23.2 (CH_2_)
11	126.3 (CH)	80.5 (CH)	123.2 (CH)	127.4 (CH)
12	137.0 (qC)	71.4 (qC)	137.1 (qC)	134.9 (qC)
13	36.4 (CH_2_)	38.9 (CH_2_)	36.6 (CH_2_)	76.9 (CH)
14	30.4 (CH_2_)	17.8 (CH_2_)	24.1 (CH_2_)	31.4 (CH_2_)
15	75.1 (qC)	134.6 (qC)	127.3 (qC)	129.9 (qC)
16	25.1 (CH_3_)	77.5 (CH_2_)	78.5 (CH_3_)	78.3 (CH_2_)
17	25.2 (CH_3_)	9.1 (CH_3_)	10.3 (CH_3_)	10.1 (CH_3_)
18	28.8 (CH_3_)	15.1 (CH_3_)	15.9 (CH_3_)	15.7 (CH_3_)
19	14.9 (CH_3_)	20.1 (CH_3_)	24.9 (CH_3_)	16.7 (CH_3_)
20	15.0 (CH_3_)	23.3 (CH_3_)	15.1 (CH_3_)	9.9 (CH_3_)
OAc			170.4 (qC)	170.3 (qC)
			20.9 (CH_3_)	21.1 (CH_3_)

*^a^* Spectra were measured in CDCl_3_ (100 MHz); *^b^* Multiplicity are deduced by HSQC and DEPT experiments.

**Figure 5:**
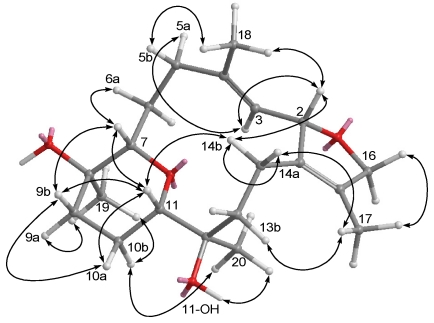
Selected NOESY correlations of **2**.

The HRESIMS of crassumol B (**2**) exhibited a pseudo molecular ion peak at *m/z*, 359.1878 ([M + Na]^+^), consistent with the molecular formula of C_2__0_H_32_O_4_, requiring five degrees of unsaturation. Its spectrum showed a broad absorption band at 3412 cm^−1^ (OH stretching). The ^13^C NMR signals at *δ*_C_ 126.8 (qC, C-1), 134.6 (qC, C-15), 126.7 (CH, C-3), and 137.6 (qC, C-4) assigned a trisubstituted double bond and a tetrasubstituted double bond in **2**, respectively. The above functionalities account for two of the five degrees of unsaturation, suggesting a tricyclic structure in **2**. Two tertiary hydroxyls were present in **2** from its ^13^C NMR signals at *δ*_C_ 68.3 (qC, C-8) and 71.4 (qC, C-12). The presence of two oxygenated methine [*δ*_H_ 3.53 (dd, 1H, *J* = 10.8, 2.8 Hz) and *δ*_C_ 83.7 (C-7); *δ*_H_ 3.74 (d, 1H, *J* = 12.0 Hz) and *δ*_C_ 80.5 (C-11)] implied that an ether linkage is probably present between C-7 and C-11, which was confirmed by the HMBC correlations from H-7 to C-11, and from H-11 to C-7. Correlations deduced from extensive analyses of the ^1^H–^1^H COSY correlations (Figure 3) of **2** enabled initially the establishment of four partial structures. The connectivity of the above structural fragments was subsequently interconnected by the HMBC correlations (Figure 3). The locations of the two double bonds at C-3/C-4 and C-1/C-15 were clarified by analysis of the HMBC correlations from Me-18 to C-3, C-4, and C-5, and from Me-17 to C-1, C-15, and C-16. Furthermore, the crucial NOE correlations (Figure 5) between H-2/Me-18, H-2/H-14b, H-11/H-7, H-11/H-9b, H-11/H-14b, H-11/H-10a, Me-19/H-9a, Me-19/H-10b, and Me-20/H-10b demonstrated the 2S*, 7S*, 8R*, 11S*, and 12R* configurations as depicted in Figure 5. Accordingly, the structure of crassumol B (**2**) was fully determined as (2S*,7S*,8R*,11S*,12R*,3*E*)-8,12-dihydroxy-7,11:2,16-bisepoxycembra-1(15),3-diene.

**Figure 6:**
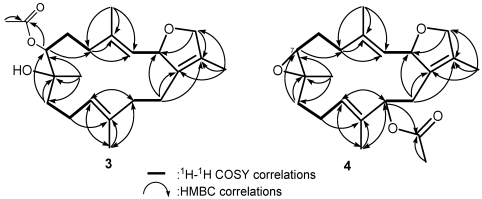
Selected ^1^H–^1^H COSY (▬) and HMBC (→) correlations of **3** and **4**.

**Figure 7:**
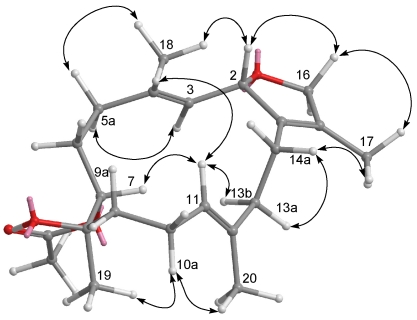
Selected NOESY correlations of **3**.

Crassumol C (**3**) was isolated as colorless oil. The HRESIMS of **3** exhibited a pseudo molecular ion peak at *m/z* 385.2854 [M + Na]^+^, consistent with the molecular formula of C_22_H_34_O_4_, implying the existence of six degrees of unsaturation. ^13^C NMR signals at *δ*_C_ 134.1 (qC, C-1), 127.3 (qC, C-15), 127.6 (CH, C-3), 137.9 (qC, C-4), 123.2 (CH, C-11), and 137.1 (qC, C-12) assigned a tetrasubstituted double bond and two trisubstituted double bonds in **3**, respectively. An acetoxymethine was present in **3** from its ^1^H NMR signals at *δ*_H_ 5.04 (1H, d, *J* = 10.0 Hz), 2.11 (3H, s) and ^13^C NMR signals at *δ*_C_ 77.3 (CH, C-7), 20.9 (CH_3_) and 170.4 (qC). The above functionalities account for four of the six degrees of unsaturation, suggesting a bicyclic structure in **3**. By interpretation of ^1^H–^1^H COSY correlations (Figure 6), it was possible to establish four partial structures of consecutive proton systems extending from H-2 to H-3, from H_2_-5 to H_2_-7 through H_2_-6, from H_2_-9 to H-11 through H_2_-10, and from H-13 to H_2_-14, as well as long-range COSY correlations between H-2/H_2_-14, H-2/H_2_-16, Me-18/H-3, and Me-17/H_2_-16. The long-range COSY correlations between H-2/H_2_-16 and Me-17/H_2_-16 also exhibited the presence of a 2,5-dihydrofuran ring in **3**. Subsequently, the connectivities of these partial structures were further established by the HMBC correlations (Figure 3). Thus, the gross structure of crassumol C was assigned as **3**. The relative stereochemistry of **3** was assigned by NOESY correlations (Figure 7). NOE correlations between H-2/Me-18, Me-18/H-11, Me-18/H-5b, H-5a/H-3, H-11/H-7, H-14a/Me-17, H-14a/H-13a, H-13b/H-11, H-10a/Me-20, H-10a/Me-19 demonstrated the 2S*, 7S*, and 8S* configurations of **3**. On the basis of the aforementioned observations, the structure of crassumol C (**3**) was unambiguously established as (2S*,7S*,8S*,3*E*,11*E*)-7-acetoxy-8-hydroxycembra-3,11-dien.

**Figure 8:**
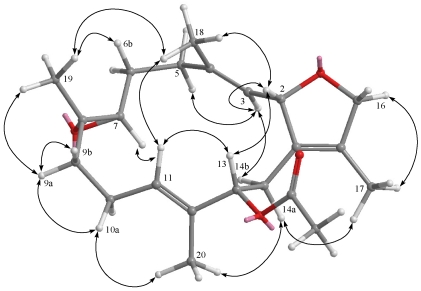
Selected NOESY correlations of **4**.

The HRESIMS of 13-acetoxysarcophytoxide (**4**) exhibited a pseudo molecular ion peak at *m/z* 383.2196 [M + Na]^+^, consistent with the molecular formula of C_2__2_H_32_O_4_, requiring seven degrees of unsaturation. A trisubstituted epoxide was recognized as being present in **4** from its ^1^H NMR signals at *δ*_H_ 2.59 (1H, t, *J* = 4.0 Hz) and ^13^C NMR signals at *δ*_C_ 59.5 (qC, C-8) and 61.7 (CH, C-7). Moreover, the ^13^C NMR signals at *δ*_C_ 130.8 (qC, C-1), 129.9 (qC, C-15), 126.1 (CH, C-3), 140.2 (qC, C-4), 127.4 (CH, C-11), and 134.9 (qC, C-12) assigned a tetrasubstituted double bond and two trisubstituted double bonds in **3**, respectively. An acetoxymethine was present in **4** from its ^1^H NMR signals at *δ*_H_ 5.16 (1H, d, *J* = 9.2 Hz), 2.00 (3H, s) and ^13^C NMR signals at *δ*_C_ 76.9 (CH, C-13), 21.1(CH_3_) and 170.3 (qC). The above functionalities account for five of the seven degrees of unsaturation, suggesting a tricyclic structure in **4**. By interpretation of ^1^H–^1^H COSY correlations (Figure 6), it was possible to establish four partial structures of consecutive proton systems extending from H-2 to H-3, from H_2_-5 to H_2_-7 through H_2_-6, from H_2_-9 to H-11 through H_2_-10, and from H-13 to H_2_-14, as well as long-range COSY correlations between H-2/H_2_-14, H-2/H_2_-16, Me-18/H-3, and Me-17/H_2_-16. The long-range COSY correlations between H-2/H_2_-16 and Me-17/H_2_-16 also exhibited the presence of a 2,5-dihydrofuran ring in **4**. Subsequently, the connectivities of these partial structures were further established by the HMBC correlations (Figure 6). The relative configurations of **4** were determined by NOE correlations (Figure 8) between H-2/Me-18, H-13/H-2, H-13/H-11, H-11/H-7, H-14a/Me-17, H-14a/Me-20, H-10a/Me-20, H-10a/H-9a, Me-18/Me-19, H-11/Me-18. On the basis of the aforementioned observations, the structure of **4** was unambiguously established as (2S*,7S*,8S*,13S*,3*E*,11*E*)-13-acetoxy-7,8-epoxycembra-3,11-dien.

The cytotoxicity against P-388 (mouse lymphocytic leukemia), HT-29 (human colon adenocarcinoma), and A-549 (human lung epithelial carcinoma) tumor cells of **1**–**4** are shown in Table 3. Compounds **1**–**4** did not show anti-HCMV activity. 

**Figure 9:**
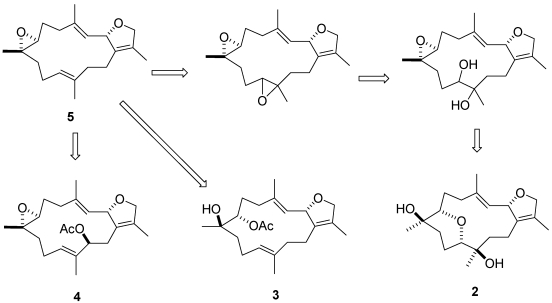
Plausible biosynthetic pathway to compounds **2**–**4**.

It is possible that compound **2** arise from the precursor with a 7,8-epoxide. Transannular opening of the 7,8-epoxide by an 11-hydroxyl would give rise to an oxepane ring in compounds **2** as shown in Figure 9. The major metabolite, (+)-sarcophytoxide (**5**), should be the precursor for compounds **2**–**4**. Therefore, the absolute configuration at C-2 for compounds **2**–**4** should be *S*.

**Table 3:** Cytotoxicity *^a^* of **1**–**4**.

Compounds	Cell lines ED_50_ (µg/mL)		
	A549	HT-29	P-388
**1**	>50	>50	>50
**2**	>50	>50	>50
**3**	>50	>50	>50
**4**	3.6	10	28
mithramycin *^b^*	0.18	0.21	0.15

*^a^* For significant activity of pure compounds, an ED_50_ of ≤4.0 µg/mL is required; *^b^* Positive control.

## 3. Experimental Section

### 3.1. General Experimental Procedures

Optical rotations were determined with a JASCO P1020 digital polarimeter. Ultraviolet (UV) and infrared (IR) spectra were obtained on a JASCO V-650 and JASCO FT/IR-4100 spectrophotometers, respectively. The NMR spectra were recorded on a Varian MR 400 NMR spectrometer at 400 MHz for ^1^H and 100 MHz for ^13^C or on a Varian Unity INOVA 500 FT-NMR spectrometer at 500 MHz for ^1^H and 125 MHz for ^13^C, respectively. Chemical shifts are expressed in *δ* (ppm) referring to the solvent peaks *δ*_H_ 7.27 and *δ*_C_ 77.0 for CDCl_3_, respectively, and coupling constants are expressed in Hz. ESI-MS were recorded by ESI FT-MS on a Bruker APEX II mass spectrometer. Silica gel 60 (Merck, Germany, 230–400 mesh) and LiChroprep RP-18 (Merck, 40–63 μm) were used for column chromatography. Precoated silica gel plates (Merck, Kieselgel 60 F_254_, 0.25 mm) and precoated RP-18 F_254s_ plates (Merck) were used for thin-layer chromatography (TLC) analysis. High-performance liquid chromatography (HPLC) was carried out using a Hitachi L-7100 pump equipped with a Hitachi L-7400 UV detector at 220 nm together with a semi-preparative reversed-phase column (Merck, Hibar LiChrospher RP-18e, 5 μm, 250 × 25 mm).

### 3.2. Biological Material

The soft coral *L.* *crassum* was collected by hand using SCUBA along the coast reefs offshore from the Dongsha Atoll in April 2007, at a depth of 6 m, and was stored in a freezer for 14 months until extraction. Identification was kindly verified by Prof. Chang-Feng Dai, Institute of Oceanography, National Taiwan University, Taiwan. A voucher specimen (TS-11) was deposited in the Department of Marine Biotechnology and Resources, National Sun Yat-sen University, Taiwan.

### 3.3. Extraction and Isolation

The frozen soft coral (2.2 kg) was chopped into small pieces and extracted exhaustively by maceration with fresh acetone for 24 h at room temperature. The quantity of solvent used for each extraction (2 L) was at least three times the amount of the soft coral material used. The acetone extracts were filtered and concentrated under vacuum to yield a brownish oily residue, which was subsequently partitioned between EtOAc and H_2_O. The resulting EtOAc-soluble residue (20.1 g) was subjected to silica gel 60 column chromatography (Si 60 CC) using hexane–EtOAc and EtOAc–MeOH of increasing polarity for elution to give roughly 40 fractions on the basis of the ^1^H NMR spectroscopic data and TLC analyses. Fraction 16 (2.0 g) eluted with *n*-hexane–EtOAc (1:2) was subjected to Si 60 CC (*n*-hexane–EtOAc, 60:1 to 1:3) to furnish 9 subfractions. A subfraction 16-4 (0.57 g) eluted with *n*-hexane–EtOAc (1:2) was subjected to a RP-18 flash column (MeOH–H_2_O, 52:48 to 100:0) to give six subfractions. In turn, a subfraction 16-4-1 (38 mg) eluted with MeOH–H_2_O (70:30) was further purified by RP-18 HPLC using MeOH–H_2_O (68:32) to give **4** (4.2 mg). Fraction 18 (2.2 g) eluted with *n*-hexane–EtOAc (1:6) was subjected to Si 60 CC (*n*-hexane–EtOAc, 20:1 to 1:3) to furnish 4 subfractions. A subfraction 18-4 (0.28 g) eluted with *n*-hexane–EtOAc (1:3) was fractionated by a RP-18 flash column (MeOH–H_2_O, 53:47 to 100:0) to give six subfractions. In turn, a subfraction 18-4-4 (35 mg) eluted with MeOH–H_2_O (80:20) was further purified by RP-18 HPLC using MeOH–H_2_O (80:20) to give **1** (3.0 mg). Subfraction 18-2 (0.11 g) eluted with *n*-hexane–EtOAc (1:1) was fractionated by a RP-18 flash column (MeOH–H_2_O, 53:47 to 100:0) to give six subfractions. In turn, a subfraction 18-2-1 (35 mg) eluted with MeOH–H_2_O (70:30) was further purified by RP-18 HPLC using MeOH–H_2_O (65:35) to give **3** (3.0 mg). Fraction 20 (0.23 g) eluted with *n*-hexane–EtOAc (1:10) was subjected to Si 60 CC (*n*-hexane–EtOAc, 1:1 to 1:10) to furnish 10 subfractions. A subfraction 20-2 (40 mg) eluted with *n*-hexane–EtOAc (1:8) was further purified by RP-18 HPLC using MeOH–H_2_O (65:35) to give **2** (2.1 mg).

Crassumol A (**1**): Colorless oil; [α]^25^_D_ −20 (*c* 0.1, CHCl_3_); IR (neat) ν_max_ 3421, 2972, 2928, 2851, 1576, 1446, 1396, 1119, 1075 cm^−1^; ^1^H NMR (CDCl_3_, 400 MHz) and ^13^C NMR (CDCl_3_, 100 MHz) data in Table 1 and Table 2; HRESIMS *m/z* 345.2408, [M + Na]^+^ (Calcd for C_2__0_H_3__4_O_3_Na, 345.2406).

Crassumol B (**2**): Colorless oil; [α]^25^_D_ −40 (*c* 0.1, CHCl_3_); IR (neat) ν_max_ 3412, 2933, 2857, 1546, 1444, 1379, 1128, 1007, 1032, 941 cm^−1^; ^1^H NMR (CDCl_3_, 400 MHz) and ^13^C NMR (CDCl_3_, 100 MHz) data in Table 1 and Table 2; HRESIMS *m/z* 359.1878 [M + Na]^+^ (Calcd for C_2__0_H_32_O_4_Na, 359.1876).

Crassumol C (**3**): Colorless oil; [α]^25^_D_ −72 (*c* 0.1, CHCl_3_); IR (neat) ν_max_ 3447, 2932, 2858, 1732, 1447, 1373, 1242, 1034 cm^−1^; ^1^H NMR (CDCl_3_, 400 MHz) and ^13^C NMR (CDCl_3_, 100 MHz) data in Table 1 and Table 2; HRESIMS *m/z* 385.2854 [M + Na]^+^ (Calcd for C_22_H_34_O_4_Na, 385.2855).

13-Acetoxysarcophytoxide (**4**): Colorless oil; [α]^25^_D_ +14 (*c* 0.1, CHCl_3_); IR (neat) ν_max_ 2939, 2858, 1733, 1663, 1446, 1373, 1237, 1042 cm^−1^; ^1^H NMR (CDCl_3_, 400 MHz) and ^13^C NMR (CDCl_3_, 100 MHz) data in Table 1 and Table 2; HRESIMS *m/z* 383.2196 [M + Na]^+^ (Calcd for C_2__2_H_32_O_4_Na, 383.2198).

### 3.4. Cytotoxicity Assay

Cytotoxicity was determined on P-388 (mouse lymphocytic leukemia), HT-29 (human colon adenocarcinoma), and A-549 (human lung epithelial carcinoma) tumor cells using a modification of the MTT colorimetric method according to a previously described procedure [11,22,23]. P-388 cell line was kindly supplied by J. M. Pezzuto, formerly of the Department of Medicinal Chemistry and Pharmacognosy, University of Illinois at Chicago. HT-29 and A-549 cell lines were purchased from the American Type Culture Collection.

### 3.5. Anti-HCMV Assay

To determine the effects of natural products upon HCMV cytopathic effect (CPE), confluent human embryonic lung (HEL) cells grown in 24-well plates were incubated for 1 h in the presence or absence of various concentrations of tested natural products. Then, cells were infected with HCMV at an input of 1000 pfu (plaque forming units) per well of 24-well dish. Antiviral activity was expressed as IC_50_ (50% inhibitory concentration), or compound concentration required to reduce virus induced CPE by 50% after 7 days as compared with the untreated control. To monitor the cell growth upon treating with natural products, an MTT-colorimetric assay was employed [24].
